# Wastewater-Based Epidemiology of Stimulant Drugs: Functional Data Analysis Compared to Traditional Statistical Methods

**DOI:** 10.1371/journal.pone.0138669

**Published:** 2015-09-22

**Authors:** Stefania Salvatore, Jørgen Gustav Bramness, Malcolm J. Reid, Kevin Victor Thomas, Christopher Harman, Jo Røislien

**Affiliations:** 1 Norwegian Centre for Addiction Research, University of Oslo, Oslo, Norway; 2 Norwegian Institute for Water Research, Oslo, Norway; 3 Department of Biostatistics, Institute of Basic Medical Sciences, Oslo, Norway; Institute for Health & the Environment, UNITED STATES

## Abstract

**Background:**

Wastewater-based epidemiology (WBE) is a new methodology for estimating the drug load in a population. Simple summary statistics and specification tests have typically been used to analyze WBE data, comparing differences between weekday and weekend loads. Such standard statistical methods may, however, overlook important nuanced information in the data. In this study, we apply functional data analysis (FDA) to WBE data and compare the results to those obtained from more traditional summary measures.

**Methods:**

We analysed temporal WBE data from 42 European cities, using sewage samples collected daily for one week in March 2013. For each city, the main temporal features of two selected drugs were extracted using functional principal component (FPC) analysis, along with simpler measures such as the area under the curve (AUC). The individual cities’ scores on each of the temporal FPCs were then used as outcome variables in multiple linear regression analysis with various city and country characteristics as predictors. The results were compared to those of functional analysis of variance (FANOVA).

**Results:**

The three first FPCs explained more than 99% of the temporal variation. The first component (FPC1) represented the level of the drug load, while the second and third temporal components represented the level and the timing of a weekend peak. AUC was highly correlated with FPC1, but other temporal characteristic were not captured by the simple summary measures. FANOVA was less flexible than the FPCA-based regression, and even showed concordance results. Geographical location was the main predictor for the general level of the drug load.

**Conclusion:**

FDA of WBE data extracts more detailed information about drug load patterns during the week which are not identified by more traditional statistical methods. Results also suggest that regression based on FPC results is a valuable addition to FANOVA for estimating associations between temporal patterns and covariate information.

## Introduction

Illicit drug use is a growing global health concern, and it is estimated that around a quarter of the European adult population has used illicit drugs at some point in their lives [1]. In Europe, central nervous system stimulants such as amphetamine and ecstasy (MDMA) are among the most commonly used illicit drugs [1]. The drugs may cause appetite suppression and euphoria with feelings of increased confidence, sociability and energy, making them popular drugs of abuse, particularly in the young [2]. Stimulant use has, however, numerous negative effects, such as insomnia, anxiety, mood disturbance, violent behaviour, dependence and psychosis making them a public health concern [3].

Because of this considerable health risk, reliable estimates of the extent of drug use in a population are important for health professionals and policy makers. Traditionally, estimates of the consumption of stimulants are calculated from data collected from sources such as treatment programmes [4], hospital emergency departments [5, 6], drivers apprehended by the police [7, 8], prisoners [9] and from population surveys (e.g., internet, population, school) [10]. These types of data, however, have their limitations, mostly related to difficulties in capturing representative survey populations. General population surveys may have poor response rates and there is often unwillingness to supply information about an activity that may have a social stigma or legal implications [10]. Further, while data from drug treatment programmes may underestimate prevalence because of limited places in treatment, data gathered from the police may overestimate prevalence as investigations are targeted towards selected populations [5–9].

Wastewater-based epidemiology (WBE) is an alternative and complementary approach for estimating the collective illicit drug use in a community [11]. The concentration of various illicit drugs in the wastewater can be measured directly, overcoming the problems related to surveys and sampling bias. WBE has shown promising results, at both local national and international level [11–13], and analyses of wastewater data have indicated differences in drug loads detected in wastewater on weekdays and at weekends [14–16]. However, as WBE is a relatively new research field, data are often analysed using simple statistical methods which do not take the temporal nature of the data fully into account, potentially overlooking important information. The aim of this study was to move beyond the simple statistical analyses often applied to wastewater-based data, in order to explore whether more advanced statistical methods can extract more information about the patterns of stimulant use.

We reanalysed a WBE dataset on 42 European cities [17] using the framework of functional data analysis (FDA), a statistical method specifically developed for analyzing temporal data [18], and we compared these results with more traditional statistical analyses. For the purpose of the study, we selected two drugs with different patterns throughout the week; ecstasy (MDMA) which is mostly a “party drug” with high expected weekend loads, and amphetamine which is expected to be used more regularly throughout the week [13]. The main temporal features for the illicit drugs throughout the course of a week were estimated using functional principal component analysis (FPCA). FPCA has recently been applied for improved statistical analysis of glucose regulation [19] and monitoring of fetal movement [20] among other things. In order to explore whether differences in temporal drug loads in the wastewater between cities could be related to various geographic or other urban characteristics, we performed both functional analysis of variance (FANOVA) as well as multiple regression analyses on the FPCA results.

## Data Material

No specific permissions were required for the present study. The use of wastewater data to study trends in illicit drug use in large catchment areas does not raise any major ethical issues as individuals cannot be identified, and thus cannot be harmed by such a study. The ethics of this approach has been thoroughly discussed in a previous paper [21].

Raw sewage was collected from the inlet of 47 sewage treatment plants in 42 cities from 21 European countries, servicing a combined population of approximately 24.7 million inhabitants. Samples were collected from each location over seven consecutive days, starting for 36 of the 42 cities on Wednesday 6th March 2013 and ending on Tuesday 12th March 2013. For the remaining six cities sampling during this week was not possible, and a different week in the same month was chosen. At all locations, automated sampling devices were used to collect subsamples over 24 hours. These subsamples were then pooled to a 24 hour composite sample. For cities with more than one sewage treatment plant, results were combined to a city average using a weighted mean. More background information, details regarding wastewater plant (WWTP) characteristics and so on can be found in an earlier publication based on the same material [17]. The data sets supporting the results of this article are also freely available [17]. For this study, we selected two drugs with very different consumption patterns; ecstasy (MDMA) which is mostly a party drug and amphetamine which is mostly used in more regular amounts throughout the week [13].

A specific tailored questionnaire was developed in cooperation with local sewage and treatment plant operators in order to evaluate information about the structure state of the sewer and the variability of the population size [17]. For the purpose of this analysis, daily mass loads were normalized by the population size of the catchment (mg/10000 people/day). Moreover concentrations for each drug below the limit of quantification (LOQ) were replaced by LOQ/2 [22] if at least one day in the week had a concentration value above the LOQ. Cities with no measurements above LOQ were excluded. Four cities (9.5%) were excluded for ecstasy (MDMA) and nine cities (21.4%) were excluded for amphetamine. Information on the characteristics of each city and WWTP is provided as supplementary material (S1 Table).

## Statistical Analysis

### Data description

The unit of observation in the analyses is a seven day week starting Wednesday and ending Tuesday. For six (14.3%) cities, the data sampling started later in the week. Missing data for the two drugs across all the 42 cities ranged from 1.7% to 2.2%. This is low [23], but since functional data analysis (FDA) requires complete datasets we performed single imputation [24] before proceeding with the analysis on the imputed dataset.

The drug loads for weekdays (Mon-Fri) and weekends (Sat-Sun) were described using median and quartiles (Q1, Q3). Wastewater drug load data was heavily skewed, and the data was log-transformed prior to further analysis.

### Traditional data analysis

For the log-transformed data for each city we calculated the overall mean throughout the week, the area under the curve (AUC) and the difference *d* between weekdays and weekends. The significance of the latter was assessed using the Wilcox test.

### Functional data analysis

The temporal pattern of wastewater drug loads was analyzed using FDA [18]. In FDA, mathematical functions are first fitted to the observations. Statistical analysis is then performed on the fitted functions rather than the original data. The seven consecutive observations for each of the 42 European cities were discrete samples of an underlying continuous process, and were converted into 42 continuous smooth curves using B-splines with seven basis functions [18, 25]. The optimal smoothing of the functions was estimated using the generalized cross validation (GCV) criterion [26] with a single choice of smoothing parameter for all cities [27]. The smoothing parameter was defined as proportion of the integrated square second derivative of the fitted curves [25]. This smoothing removes the random day-to-day variation, e.g. non-systematic error, measurement error and normal fluctuations in the load of drugs, and so extracts the underlying temporal behaviour.

### Functional principal component analysis

Principal component analysis (PCA) is a statistical methodology which is used to reveal the internal structure of the data in order to explain variability [28]. Functional principal component analysis (FPCA) is a generalization of traditional PCA to functional data [18]. A common practice in FPCA is to first normalize the data, that is, to first subtract the mean, as the mean curve is a mode of variation that tends to be shared by most curves [25]. However, as we were interested in the mean temporal differences in drug loads between cities, and also compare to traditional statistical methods, we did not normalize the data. The percentage of explained variation for each FPCs thus cannot be interpreted in the same way as for PCA on normalized data.

We used FPCA to identify the main temporal patterns across the 42 fitted smooth curves. The result of an FPCA is a set of mutually uncorrelated functional principal component (FPC) curves, which explain the main modes of temporal variability across the fitted curves for all cities. The analysis further provides each city with a score for each of the extracted FPC curves, representing the intensity with which that particular temporal pattern is present in the fitted function for that particular city. Cities with close to zero scores on all FPCs have temporal drug loads similar to the overall mean curve, while cities with a high score on a particular FPC have a temporal drug load closer to that specific FPC pattern. Each estimated FPC was interpreted and labelled according to the temporal information it exhibited.

The association between the more traditional statistical measures of wastewater drug loads and the FPCA was assessed by calculating the Pearson correlation coefficient (r) between the FPC scores, the overall mean of the log-transformed data, AUC and the difference *d* between weekdays and weekend means.

### Functional analysis of variance

To move beyond mere exploration of patterns, we wanted to see whether the various temporal patterns of wastewater drug loads throughout a week were associated with basic characteristics of the city: latitude; longitude; gross domestic product (GDP) of country; relative size of the city, i.e., number of inhabitants in the city divided by the number of inhabitants in the country and density of the city, i.e., number of inhabitants in the city divided by the urban area of the city.

Traditional analysis of variance (ANOVA) explores the mean difference in a continuous response between the various categories in a categorical explanatory variable [29]. Functional analysis of variance (FANOVA) is the generalization of ANOVA to functional outcomes [18], and is often the suggested approach when exploring covariates in FDA [18]. We used FANOVA to analyze the effect of the five possible predictors, listed above, on the shape of the wastewater drug load curves [25]. As FANOVA must have categorical covariates we had to dichotomize each of the continuous explanatory variables and compared the mean curves in the two resulting groups. We explored the impact of cut-off point by selecting cut-off points across the whole observed range of the covariates, and considered p-values <0.05 to be statistically significant. Functional confidence intervals (95%) and p-value curves, as well as an overall p-value, were calculated for each covariate using a functional permutation F-test [25].

### Multiple regression

FANOVA can be seen as a univariate ANOVA problem for each specific point in time [18], and thus cannot control for covariates. In order to explore multiple predictors simultaneously, without the need for dichotomization, we used the cities' scores for the estimated FPCs as outcome variables in multiple linear regression models. From the full multiple model, including all covariates, an optimal sub-model was chosen using Akaike's Information Criterion (AIC) [30]. AIC is a weighting between model parsimony and fit to the data, and is a measure of the "goodness" of a model [31], and can be used to compare statistical models.

### Robustness analysis

To explore the robustness of the FDA results, i.e. whether temporal patterns would emerge purely by chance due to the nature of the curve fitting process, we also performed all of the above FDA on a dataset obtained by random sorting of the original data.

### Software

All analyses were performed in R 3.1.0 [32]. The imputation was performed using Amelia II and the amelia package [33], and FDA, FPCA and FANOVA using the fda package [25].

## Results

### Data summary

Wastewater drug loads for the 42 cities throughout the week are shown in Fig 1.1 and 1.2, and summarized in Table 1. The median load of ecstasy (MDMA) increased significantly at the weekend (p<0.001) but not for amphetamine (p = 0.369). The overall mean of the log-transformed data throughout the seven day week was highly correlated (r = 0.999) with the area under the curve (AUC) (Tables 2 and 3).

**Fig 1:**
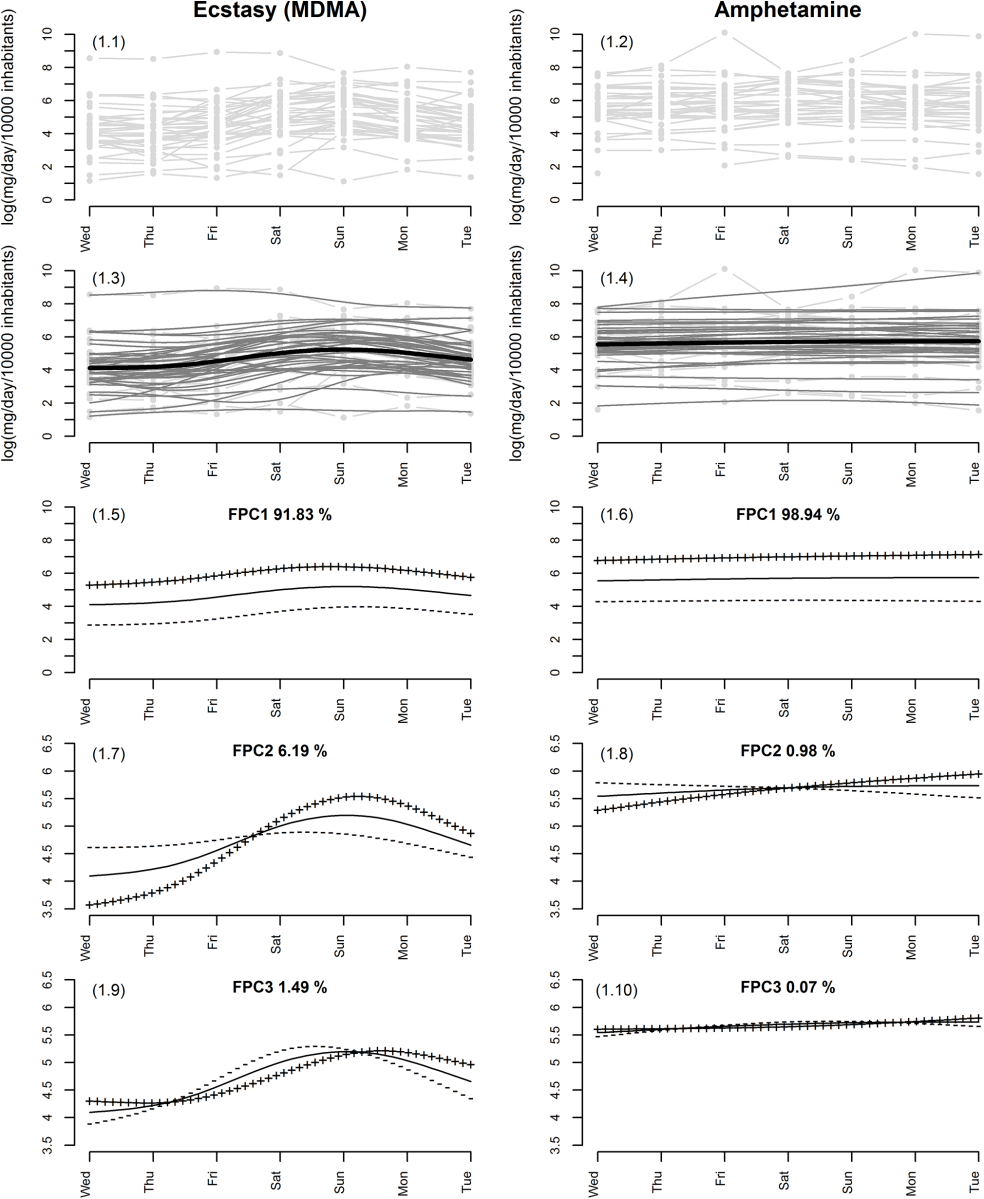
Raw data, individual curves and results from the FPCA for each drug. 1.1–1.2 shows the raw data for each drug; 1.3–1.4 shows the raw data (light grey) with the individually fitted curves (dark grey) and the mean of these curves (black); 1.5–1.10 shows the mean of the fitted curves (solid line) and how the shape of an individual curve differs from the mean curve if a multiple of the principal component curve is added to (+ +) or subtracted from (- -) the mean curve. The multiples correspond to one SD of the FPC1, FPC2 and FPC3 scores, respectively.

**Table 1:** Wastewater drug loads for 42 European cities throughout the week.

Day	Ecstasy (MDMA) *		Amphetamine**	
	Median	Q1, Q3	Median	Q1, Q3
Wednesday	5.7	3.4, 12.7	23.4	13.4, 53.5
Thursday	6.8	2.6, 11.8	29.8	15.5, 65.1
Friday	8.5	4.2, 20.3	30.5	14.8, 72.6
*Saturday*	*15*.*9*	*5*.*9*, *45*.*7*	*30*.*2*	*16*.*6*, *91*.*0*
*Sunday*	*20*.*2*	*10*.*5*, *54*.*8*	*34*.*0*	*17*.*4*, *68*.*8*
Monday	14.1	5.8, 26.9	31.3	16.9, 69.3
Tuesday	9.9	5.0, 17.9	28.3	16.2, 64.0

*Statistically significant difference between weekday (Mon-Fri) and weekend (Sat-Sun) loads using the Wilcox test (p-value < 0.001)

**No statistically significant difference between weekday (Mon-Fri) and weekend (Sat-Sun) loads using the Wilcox test

(p-value = 0.369)

The data sets supporting the table are freely available [17].

**Table 2:** Pearson correlation coefficients between FPC scores for the ecstasy (MDMA) loads and simple summary measures.

	Simple summary measures			FPCA scores		
	Mean*	d**	AUC	FPC1	FPC2	FPC3
Mean*	1.000					
d**	0.019	1.000				
AUC	0.999	0.044	1.000			
FPC1	0.999	0.044	0.999	1.000		
FPC2	-0.005	0.762	0.009	0.000	1.000	
FPC3	0.038	-0.497	0.013	0.000	0.000	1.000

*Overall mean of the log-transformed data throughout the seven day week.

**Difference: mean of the log-transformed data (weekend) minus mean of the log-transformed data (weekdays).

**Table 3:** Pearson correlation coefficients between FPC scores for amphetamine loads and simple summary measures.

	Simple summary measures			FPCA scores		
	Mean*	d**	AUC	FPC1	FPC2	FPC3
Mean*	1.000					
d**	-0.267	1.000				
AUC	0.999	-0.256	1.000			
FPC1	0.999	-0.262	0.999	1.000		
FPC2	-0.005	-0.217	0.003	0.000	1.000	
FPC3	0.009	-0.760	-0.011	0.000	0.000	1.000

*Overall mean of the log-transformed data throughout the seven day week.

**Difference: mean of the log-transformed data (weekend) minus mean of the log-transformed data (weekdays).

### Functional principal component analysis

The original data with the corresponding fitted smooth curves of log-transformed data are shown in Fig 1.3 and 1.4.

For both drugs, the first functional principal component (FPC1; representing the normalization of the original data) explained more than 90% of the total temporal variation between the fitted curves for the cities (Fig 1.5 and 1.6), while the three first FPCs in sum explained more than 99% of the total variation (Fig 1.5–1.10). For ecstasy (MDMA) the first FPC (FPC1) mainly represented the general level, including a small increase towards the second half of the weekend (Fig 1.5). The second FPC (FPC2) represented an additional increase in the second half of the weekend (Fig 1.7), and the third FPC (FPC3) the timing of this weekend peak (Fig 1.9). For amphetamine, FPC1 represented the general level and this alone explained 98.9% of the variance (Fig 1.6). FPC2 represented a miniscule, linear increase throughout the week (Fig 1.8) and FPC3 an increase at the weekend (Fig 1.10).

Pearson's correlation between the simple summary measures and the scores for the first three FPCs for ecstasy (MDMA) and amphetamine are shown in Tables 2 and 3. For ecstasy (MDMA), the mean week load and the AUC statistics were almost perfectly correlated with FPC1 (r = 0.999), while the difference between weekday and weekend loads was moderately correlated with FPC2 (r = 0.762) and FPC3 (r = -0.497). For amphetamine, the mean week load and the AUC statistics were almost perfectly correlated with FPC1 (r = 0.999), while the difference between weekday and weekend loads was moderately correlated with FPC3 (r = -0.760). The rest of the correlations were miniscule.

### Functional analysis of variance

**Fig 2:**
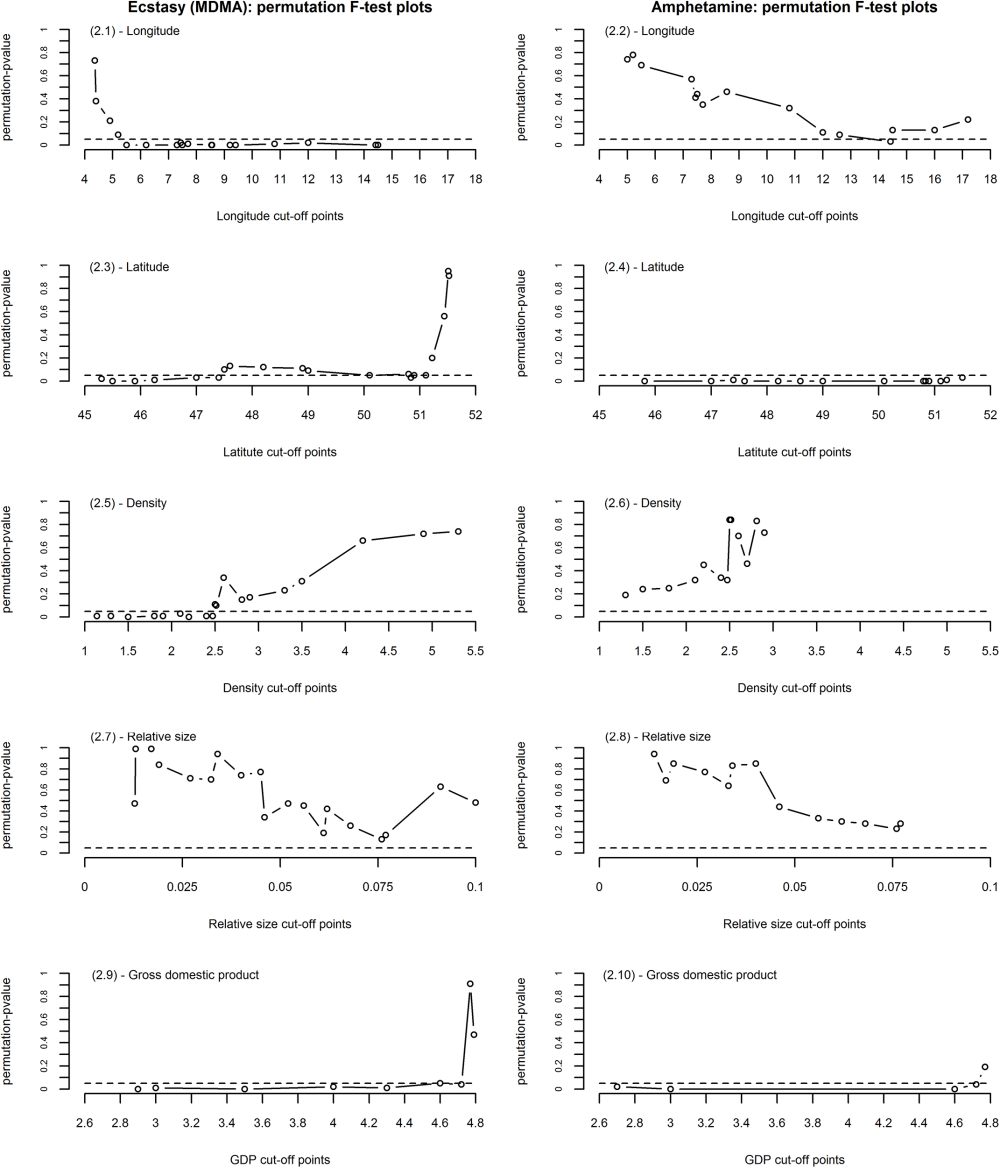
FANOVA F-permutation test plot separately for each drug and for each explanatory variable. 2.1–2.2 show how the p-value of the permutation F-test changes, as different values of longitude are chosen as grouping; 2.3–2.4 show how the p-value of the permutation F-test changes, as different values of latitude are chosen as grouping; 2.5–2.6 show how the p-value of the permutation F-test changes, as different values of density are chosen as grouping; 2.7–2.8 show how the p-value of the permutation F-test changes, as different values of relative size are chosen as grouping; 2.9–2.10 show how the p-value of the permutation F-test changes, as different values of gross domestic product (GDP) are chosen as grouping.

The functional analysis of variance (FANOVA) showed that latitude, longitude and gross domestic product (GDP) were all potentially associated with ecstasy (MDMA) load throughout the week, depending on the cut-off value, while only latitude and GDP were associated with the mean load curve for amphetamine. For some of the predictors, choice of cut-off value for the dichotomization had a major impact on the estimated significance of the difference between the mean of the corresponding groups (Fig 2).

### Multiple regression analyses

For ecstasy (MDMA), multiple linear regression using FPC scores as outcome showed that longitude was negatively associated with scores on FPC1, i.e. the general level of the ecstasy load in the wastewater tended to increase in a westerly direction, while latitude was positively associated with scores on FPC1, i.e. the level of the ecstasy load tended to increase in a northerly direction (Table 4). The longitude of the city was also negatively associated with scores on FPC2, i.e. more pronounced weekend peaks were observed in a westerly direction.

**Table 4:** Multiple regression analyses with functional principal component scores for ecstasy (MDMA) as dependent variable and longitude, latitude, gross domestic product, population density and relative size of the city as explanatory variables.

	FPC1 Scores				FPC2 Scores				FPC3 Scores			
	Multiple Model(AIC* 194.58)		Optimal Model(AIC* 189.54)		Multiple Model(AIC* 98.30)		Optimal Model(AIC* 94.19)		Multiple Model(AIC* 44.52)		Optimal Model(AIC* 40.22)	
	Estimate(95% CI)	p-value	Estimate(95% CI)	p-value	Estimate(95% CI)	p-value	Estimate(95% CI)	p-value	Estimate(95% CI)	p-value	Estimate(95% CI)	p-value
Longitude	-0.155(-0.258,-0.052)	0.006	-0.161(-0.250,-0.071)	0.001	-0.029(-0.059,0.001)	0.065	-0.026(-0.052,0.001)	0.068	0.007(-0.008,0.022)	0.390		
Latitude	0.122(-0.026,0.269)	0.115	0.130(0.009,0.251)	0.042	0.032(-0.011,0.075)	0.150			0.003(-0.019,0.024)	0.821		
Gross domestic product ^a^	0.137(-0.307,0.581)	0.549			-0.038(-0.167,0.092)	0.572			-0.040(-0.105,0.024)	0.230		
Population density ^b^	0.044(-0.148,0.237)	0.654			0.024(-0.032,0.080)	0.419			0.017(-0.011,0.045)	0.255		
Size of city ^c^	2.414(-11.975,16.804)	0.744			1.903(-2.285,6.091)	0.380			-2.082(-4.184,0.020)	0.061		

* Akaike's information criterion.

a Number taken from http://en.wikipedia.org/wiki/List_of_countries_by_GDP_%28nominal%29_per_capita.

b Number of inhabitants in city divided by the urban area in square kilometres.

c Number of inhabitants in city divided by the number of inhabitants in the country.

For amphetamine, multiple linear regression using FPC scores as outcome showed that latitude was positively associated with scores on FPC1, i.e. the level of the amphetamine load in the wastewater tended to increase in a northerly direction. Latitude was also negatively associated with the scores on FPC3, i.e. more pronounced weekend peaks were observed in a northerly direction (Table 5).

**Table 5:** Multiple regression analyses with functional principal component scores for amphetamine as dependent variable and longitude, latitude, gross domestic product, population density and size of the city as explanatory variables.

	FPC1 Scores				FPC2 Scores				FPC3 Scores			
	Multiple Model(AIC* 173.69)		Optimal Model(AIC* 168.87)		Multiple Model(AIC* 30.71)		Optimal Model(AIC* 23.61)		Multiple Model(AIC* -63.32)		Optimal Model(AIC* -67.62)	
	Estimate(95% CI)	p-value	Estimate(95% CI)	p-value	Estimate(95% CI)	p-value	Estimate(95% CI)	p-value	Estimate(95% CI)	p-value	Estimate(95% CI)	p-value
Longitude	-0.082(-0.221,0.057)	0.256	-0.074(-0.178,0.030)	0.175	-0.005(-0.022,0.012)	0.584			0.004(-0.0004,0.008)	0.089		
Latitude	0.295(0.124,0.465)	0.002	0.255(0.121,0.389)	<0.001	0.002(-0.019,0.023)	0.861			-0.005(-0.010,0.0001)	0.064	-0.004(-0.009,-0.0004)	0.039
Gross domestic product ^a^	-0.105(-0.623,0.368)	0.693			-0.044(-0.107,0.019)	0.185	-0.034(-0.082,0.014)	0.172	0.005(-0.011,0.021)	0.575		
Population density ^b^	0.123(-0.142,0.384)	0.368			-0.008(-0.040,0.024)	0.610			-0.0003(-0.008,0.008)	0.938		
Size of city ^c^	1.749(-16.408,19.907)	0.852			0.049(-2.169,2.267)	0.993			-0.314(-0.870,0.242)	0.278		

* Akaike's information criterion.

a Number taken from http://en.wikipedia.org/wiki/List_of_countries_by_GDP_%28nominal%29_per_capita.

b Number of inhabitants in city divided by the urban area in square kilometres.

c Number of inhabitants in city divided by the number of inhabitants in the country.

### Robustness analysis

For the randomly sorted data, the smoothing parameter for both drugs was the local maximum of the chosen interval, indicating that all the variability from the data was composed by random variation.

The first FPC explained 64–71% of the total temporal variation between the fitted curves, while the second and third FPCs explained 30–36% and 0% of the total variation respectively. Neither of the FPCs showed any specific pattern. Moreover, the functional permutation F-test of the FANOVA analysis was not able to distinguish between groups of curves for either of the drugs (not shown).

## Discussion

The objective of wastewater-based epidemiology (WBE) is to provide objective and reliable estimates of the abuse of various drugs within a population. It is a relatively new methodology within the health sciences, but has already shown promising results [11–17], and promises to be a valuable addition to more traditional data sources. How to best analyse such data is, however, unclear.

As in many medical research fields, the analysis of WBE data tends to be performed by researchers with their primary field of expertise outside of statistics, and WBE data have generally been analyzed using traditional statistical methods, such as simple summary measures and specification tests [13, 15], which focus only on level of use [13, 17]. Simple statistical methods have the advantage that they are easily understood and performed by most quantitative scientists. Such methods are, however, problematic if they do not utilize the data properly or, worse, lead to the wrong conclusions. Using traditional statistical methods and specification tests we were not able to identify any weekend pattern for amphetamine throughout the week, but we were able to demonstrate this using functional principal component analysis (FPCA).

Understanding temporal patterns of stimulant drug consumption could help us to understand the extent of illicit drug problems better and suggest more effective preventive actions. This study is the first to use the framework of FDA to extract shape information from wastewater-based drug load data. While the mean of the fitted curves obtained from FDA represents information about the use of these two substances across Europe which was already known [13, 17] using FDA, and in particular FPCA, we were also able to extract valuable, nuanced temporal information on the use of stimulant drugs throughout the week that simpler statistical methods missed.

FPCA decomposes the variation between curves into a set of uncorrelated temporal features, but the usefulness of this analysis depends on how the FPCs are interpreted. In our study, FPC1 mainly represented the general drug load, accounting alone for more than 90% of the temporal variability between cities. Interestingly, AUC was almost perfectly correlated with the FPC1 for both drugs, demonstrating that AUC carries both valuable and precise information about an important part of the temporal drug load.

The second and third FPCs roughly represented how pronounced a weekend peak was and the timing of such a peak. Even though they account for only 0.1–6.2% of the temporal variability, they paint a more nuanced picture of the drug use pattern that would be lost when using traditional statistical analyses. The difference *d* between weekday and weekend means was somewhat correlated with FPC2, but neither of the simple summary measures can be said to capture fully the information in the FPCs beyond FPC1.

Our results suggest that even when considering a drug with a smoothed behaviour throughout the week such as amphetamine, FPCA is able to capture difference in variability between weekdays and weekends. Moreover, since the second and third FPCs are uncorrelated, our analysis was able to untangle the part of the variability mainly due to the increasing drug load at the weekend and the timing of such an increase. FDA results also demonstrate that the “weekend” is a somewhat less well defined time period than the traditional cultural understanding of it. Using this approach one may estimate what constitutes the “weekend” for each city and each drug, without having to define it a priori, as is needed when applying standard statistical tests.

Performing multiple regression analyses using FPC scores as outcome variables, we found that the temporal patterns were associated with the geographical position of the city; the load of ecstasy increased significantly in north-west Europe, while the load of amphetamine increased in a northerly direction. This is generally in line with previous findings [34].

Usually, FANOVA is the suggested way to analyse the association between functional data and covariates [18]. However, FANOVA needs dichotomous explanatory variables, and most of the predictors that we investigated were continuous. Categorizing continuous predictors in regression models has been thoroughly examined in the statistical literature, and repeatedly argued against, as it reduces power and introduces bias of unknown direction and magnitude [35–37]. In our study applying FANOVA would introduce bias in the analysis, due to the arbitrary choice of the city groups [35]. The significance of the F-test strongly depended on the chosen cut-off level of the explanatory variable. Moreover, FANOVA cannot adjust for other covariates in a multiple regression model and it only looks at the mean temporal pattern. While this will verify differences between cities, it will not identify the mode of the difference. Our suggested multiple regression is not part of the original FDA framework, but opens for more flexibility. It has been proposed previously for the analysis of glucose and fetal movement data [19, 20].

FDA shows several advantages over traditional approaches to analysing temporal data from wastewater treatment plants, and should be explored for drugs other than the two stimulant drugs we studied here. However, a major issue in the application of FPCA is the ability to interpret the patterns shown by the most important FPCs, so as to consider appropriate predictors to explain them. Also, our use of FANOVA as the sole means of introducing covariate information in FDA regressions could be explored further.

FDA is developed for analysing temporal data, but a potential concern is that the smooth basis functions applied in FDA run the risk of smoothing over more abrupt changes in the drug load throughout the week, consequently underestimating for example the difference between week and weekend load. Wavelets have a long tradition in time series analysis, and allows for statistical modelling of less smooth temporal changes. Wavelet based PCA has recently been applied successfully to among others fetal movement data [38, 39] and a similar approach might be worthwhile exploring also for WWA data.

Other future research should include more information about the structure of the sewage system, as well as longer periods of observation and more cities to further improve the statistical analysis, monitor the temporal variation and achieve a better overall picture of the use of illicit drugs in Europe.

## Supporting Information

S1 Table Summary of information of participating cities. (DOC)

## References

[1] EMCDDA. European Drug Report: Trends and developments. 2013.

[2] Tossmann P, Boldt S, Tensil MD. The use of drugs within the techno party scene in European metropolitan cities. Eur Addict Res. 2001;7(1):2–23. 10.1159/000050709 . 11316921

[3] Hartel-Petri R, Rodler R, Schmeisser U, Steinmann J, Wolfersdorf M. Increasing prevalence of amphetamine- and methamphetamine-induced psychosis—Regional frequency in upper francomial bavaria. Psychiatr Prax. 2005;32(1):13–7. 10.1055/s-2003-814996 . 15633070

[4] Lyne J, O'Donoghue B, Clancy M, Kinsella A, O'Gara C. Concurrent cocaine and alcohol use in individuals presenting to an addiction treatment program. Ir J Med Sci. 2010;179(2):233–7. 10.1007/s11845-009-0385-6 . 19597917

[5] Kerr T, Wood E, Grafstein E, Ishida T, Shannon K, Lai C, et al High rates of primary care and emergency department use among injection drug users in Vancouver. J Public Health (Oxf). 2005;27(1):62–6. 10.1093/pubmed/fdh189 . 15564279

[6] Bruggisser M, Ceschi A, Bodmer M, Wilks MF, Kupferschmidt H, Liechti ME. Retrospective analysis of stimulant abuse cases reported to the Swiss Toxicological Information Centre during 1997–2009. Swiss Med Wkly. 2010;140:w13115 10.4414/smw.2010.13115 . 21188679

[7] Stig Tore Bogstrand GM, Asbjørg S. Christophersen. Trends in amphetamine and benzodiazepine use among drivers arrested for drug impaired driving in Norway 2000–2009. Norwegian Journal of Epidemiology. 2011;Vol 21, No 1 (2011)

[8] Legrand SA, Houwing S, Hagenzieker M, Verstraete AG. Prevalence of alcohol and other psychoactive substances in injured drivers: comparison between Belgium and The Netherlands. Forensic Sci Int. 2012;220(1–3):224–31. 10.1016/j.forsciint.2012.03.006 . 22483531

[9] Farrell M, Marsden J. Acute risk of drug-related death among newly released prisoners in England and Wales. Addiction. 2008;103(2):251–5. 10.1111/j.1360-0443.2007.02081.x . 18199304

[10] Smith GW, Farrell M, Bunting BP, Houston JE, Shevlin M. Patterns of polydrug use in Great Britain: Findings from a national household population survey. Drug Alcohol Depend. 2011;113(2–3):222–8. 10.1016/j.drugalcdep.2010.08.010 . 20863629

[11] Zuccato E, Chiabrando C, Castiglioni S, Bagnati R, Fanelli R. Estimating community drug abuse by wastewater analysis. Environ Health Perspect. 2008;116(8):1027–32. 10.1289/ehp.11022 18709161 PMC2516581

[12] van Nuijs AL, Castiglioni S, Tarcomnicu I, Postigo C, Lopez de Alda M, Neels H, et al Illicit drug consumption estimations derived from wastewater analysis: a critical review. The Science of the total environment. 2011;409(19):3564–77. 10.1016/j.scitotenv.2010.05.030 . 20598736

[13] Thomas KV, Bijlsma L, Castiglioni S, Covaci A, Emke E, Grabic R, et al Comparing illicit drug use in 19 European cities through sewage analysis. The Science of the total environment. 2012;432:432–9. 10.1016/j.scitotenv.2012.06.069 . 22836098

[14] van Nuijs AL, Mougel JF, Tarcomnicu I, Bervoets L, Blust R, Jorens PG, et al A one year investigation of the occurrence of illicit drugs in wastewater from Brussels, Belgium. J Environ Monit. 2011;13(4):1008–16. 10.1039/c0em00686f . 21331424

[15] Reid MJ, Langford KH, Morland J, Thomas KV. Quantitative assessment of time dependent drug-use trends by the analysis of drugs and related metabolites in raw sewage. Drug Alcohol Depend. 2011;119(3):179–86. 10.1016/j.drugalcdep.2011.06.007 . 21737215

[16] Kinyua J, Anderson TA. Temporal Analysis of the Cocaine Metabolite Benzoylecgonine in Wastewater to Estimate Community Drug Use. J Forensic Sci. 2012;57(5):1349–53. 10.1111/j.1556-4029.2012.02135.x . 22509844

[17] Ort C, van Nuijs AL, Berset JD, Bijlsma L, Castiglioni S, Covaci A, et al Spatial differences and temporal changes in illicit drug use in Europe quantified by wastewater analysis. Addiction. 2014 10.1111/add.12570 . 24861844 PMC4204159

[18] Ramsay JO SB. Functional data analysis: Springer; 2005.

[19] Froslie KF, Roislien J, Qvigstad E, Godang K, Bollerslev J, Voldner N, et al Shape information from glucose curves: functional data analysis compared with traditional summary measures. BMC Med Res Methodol. 2013;13:6 10.1186/1471-2288-13-6 23327294 PMC3570313

[20] Winje BA, Roislien J, Froen JF. Temporal patterns in count-to-ten fetal movement charts and their associations with pregnancy characteristics: a prospective cohort study. BMC Pregnancy Childbirth. 2012;12:124 10.1186/1471-2393-12-124 23126608 PMC3542088

[21] Prichard J, Hall W, de Voogt P, Zuccato E. Sewage epidemiology and illicit drug research: the development of ethical research guidelines. The Science of the total environment. 2014;472:550–5. 10.1016/j.scitotenv.2013.11.039 . 24317162

[22] Ort C, Lawrence MG, Rieckermann J, Joss A. Sampling for Pharmaceuticals and Personal Care Products (PPCPs) and Illicit Drugs in Wastewater Systems: Are Your Conclusions Valid? A Critical Review. Environ Sci Technol. 2010;44(16):6024–35. 10.1021/Es100779n . 20704196

[23] Scheffer J. Dealing with missing data. 2002.

[24] Rubin DD. Multiple imputation for nonresponse in surveys. New York: J; 1987.

[25] Ramsay JO, Hooker G, Graves S. Functional data analysis with R and MATLAB Dordrecht; New York: Springer; 2009 xi, 207 p. p.

[26] Craven P, Wahba G. Smoothing noisy data with spline functions. Numerische Mathematik. 1978;31(4):377–403.

[27] Silverman BW. Smoothed functional principal components analysis by choice of norm. The Annals of Statistics. 1996;24(1):1–24.

[28] Jolliffe IT. Principal component analysis 2nd ed. New York: Springer; 2002 xxix, 487 p. p.

[29] Mertler CA, Vannatta RA. Advanced and multivariate statistical methods Los Angeles, CA: Pyrczak 2002.

[30] Akaike H. New Look at Statistical-Model Identification. Ieee Transactions on Automatic Control. 1974;Ac19(6):716–23. 10.1109/Tac.1974.1100705 .

[31] Burnham KP, Anderson DR. Model selection and multimodel inference: a practical information-theoretic approach: Springer; 2002.

[32] Team RC. The R Foundation for Statistical Computing: R version 3.1.0 (2014.04.05). Available: http://www.r-project.org2014.

[33] Honaker J, King G, Blackwell M. Amelia II: A Program for Missing Data. Journal of Statistical Software. 2011;45(7):1–47. .

[34] EMCDDA. The state of the drugs problem in Europe. 2010.

[35] Royston P, Altman DG, Sauerbrei W. Dichotomizing continuous predictors in multiple regression: a bad idea. Statistics in medicine. 2006;25(1):127–41. 10.1002/sim.2331 . 16217841

[36] Altman DG, Royston P. The cost of dichotomising continuous variables. Bmj. 2006;332(7549):1080 10.1136/bmj.332.7549.1080 16675816 PMC1458573

[37] van Walraven C, Hart RG. Leave 'em alone—Why continuous variables should be analyzed as such. Neuroepidemiology. 2008;30(3):138–9. 10.1159/000126908 . 18421216

[38] Roislien J, Winje B. Feature extraction across individual time series observations with spikes using wavelet principal component analysis. Statistics in medicine. 2013;32(21):3660–9. 10.1002/sim.5797 . 23553851

[39] Winje BA, Roislien J, Saastad E, Eide J, Riley CF, Stray-Pedersen B, et al Wavelet principal component analysis of fetal movement counting data preceding hospital examinations due to decreased fetal movement: a prospective cohort study. BMC Pregnancy Childbirth. 2013;13:172 10.1186/1471-2393-13-172 24007565 PMC3844562

