# Supplementary material for: Wastewater-Based Epidemiology of Stimulant Drugs: Functional Data Analysis Compared to Traditional Statistical Methods
Source: PLoS One. 2015 Sep 22;10(9):e0138669. doi: 10.1371/journal.pone.0138669 (PMC4578919; doi:10.1371/journal.pone.0138669)
Supplement: S1 Table — (DOC) [file pone.0138669.s001.doc]

**S1 Table – *Summary of information of participating cities**

| *Country*** | *City* | *Inhabitant country* | *Inhabitant city* | *Density* | *Gross Domestic Product (GDP) per capita $* | *Latitude* | *Longitude* |
| --- | --- | --- | --- | --- | --- | --- | --- |
| BA | Sarajevo | 3 871 643 | 369 534 | 2611 | 4 865 | 43.87 | 18.42 |
| BE | Antwerp Deurne | 11 099 554 | 507 368 | 2479 | 45 383 | 51.22 | 4.38 |
|  | Antwerp Zuid | 11 099 554 | 507 368 | 2479 | 45 383 | 51.22 | 4.38 |
|  | Brussels | 11 094 850 | 1 159 448 | 7025 | 45 383 | 51.63 | 4.50 |
|  | Geraardsbergen | 11 094 850 | 32 629 | 410 | 45 383 | 50.77 | 3.87 |
|  | Koksijde | 11 094 850 | 22 153 | 500 | 45 383 | 51.10 | 2.65 |
|  | Ninove | 11 094 850 | 37 295 | 720 | 45 383 | 50.83 | 4.02 |
| CH | Basel | 7 954 662 | 310 395 | 7619 | 81 323 | 47.57 | 7.60 |
|  | Berne | 7 954 662 | 212 891 | 2471 | 81 323 | 46.95 | 7.45 |
|  | Geneva | 7 954 662 | 350 924 | 12261 | 81 323 | 46.20 | 6.15 |
|  | St.Gallen (2 WWTPs) | 7 954 662 | 73 505 | 1880 | 81 323 | 47.42 | 9.37 |
|  | Zurich | 7 954 662 | 598 986 | 4176 | 81 323 | 47.37 | 8.55 |
| CY | Nicosia | 1 117 000 | 310 355 | 2800 | 26 389 | 35.17 | 33.37 |
|  | Limassol | 1 117 000 | 101 000 | 35 | 26 389 | 34.67 | 33.03 |
| CZ | Budweis | 10 516 125 | 93 467 | 1729 | 18 857 | 49.40 | 14.97 |
|  | Prague | 10 516 125 | 1 246 780 | 2513 | 18 857 | 50.08 | 14.42 |
| DE | Dortmund | 81 843 743 | 580 956 | 2000 | 47 893 | 51.52 | 7.43 |
|  | Dülmen | 81 843 743 | 46 071 | 250 | 47 893 | 52.67 | 7.97 |
|  | Dresden | 81 843 743 | 529 781 | 1600 | 47 893 | 51.03 | 13.73 |
| DK | Copenhagen | 5 602 628 | 559 440 | 6600 | 59 190 | 56.23 | 12.67 |
| ES | Barcelona | 47 265 321 | 3 202 571 | 15991 | 29 150 | 41.38 | 2.17 |
|  | Castellon | 47 265 321 | 604 564 | 91 | 29 150 | 40.17 | -0.17 |
|  | Santiago | 47 265 321 | 95 671 | 429 | 29 150 | 43.82 | 9.01 |
|  | Valencia (3 WWTPs) | 47 265 321 | 797 028 | 6000 | 29 150 | 39.48 | 0.37 |
| FI | Helsinki | 5 401 267 | 1 059 631 | 2873 | 47 129 | 60.42 | 25.18 |
|  | Turku | 5 401 267 | 178 630 | 742 | 47 129 | 60.53 | 22.27 |
| *Country*** | *City* | *Inhabitant country* | *Inhabitant city* | *Density* | *Gross Domestic Product (GDP) per capita $* | *Latitude* | *Longitude* |
| FR | Paris | 66 616 416 | 10 413 386 | 21000 | 42 999 | 49.25 | 2.47 |
| GB | London | 63 705 000 | 8 308 369 | 5285 | 39 567 | 51.93 | -0.77 |
| GR | Athens | 10 816 286 | 664 064 | 17043 | 21 617 | 37.97 | 23.72 |
| HR | Zagreb | 4 284 889 | 792 875 | 1200 | 13 561 | 45.80 | 15.97 |
| IT | Milan | 59 943 933 | 3 105 489 | 7237 | 34 714 | 46.30 | 9.60 |
| NL | Amsterdam | 16 730 348 | 1 021 754 | 4892 | 47 633 | 52.37 | 4.87 |
|  | Eindhoven | 16 730 348 | 217 225 | 2517 | 47 633 | 51.57 | 6.30 |
|  | Utrecht | 16 730 348 | 316 275 | 3483 | 47 633 | 52.42 | 6.03 |
| NO | Oslo | 4 985 870 | 613 285 | 1400 | 100 318 | 59.92 | 10.75 |
| PT | Lisbon | 10 542 398 | 1 860 256 | 6458 | 21 765 | 38.77 | -9.15 |
| RO | Cluj Napoca | 20 121 641 | 324 576 | 1808 | 8 775 | 46.77 | 23.58 |
| RS | Belgrade | 7 186 862 | 1 166 763 | 3241 | 5 906 | 44.82 | 20.47 |
|  | Novi Sad | 7 186 862 | 231 798 | 2182 | 5 906 | 45.25 | 19.85 |
| SE | Gothenburg | 9 658 301 | 533 260 | 1200 | 57 909 | 57.70 | 11.97 |
|  | Umeå | 9 658 301 | 79 594 | 2331 | 57 909 | 63.83 | 20.92 |
| SK | Bratislava (2 WWTPs) | 5 410 836 | 415 589 | 1131 | 18 792 | 48.77 | 17.68 |
|  | Piestany | 5 410 836 | 29 660 | 671 | 18 792 | 48.63 | 17.85 |

*Number taken from ([http://epp.eurostat.ec.europa.eu](http://epp.eurostat.ec.europa.eu/); <http://en.wikipedia.org/wiki>)

**BA – Bosnia Herzegovina, BE – Belgium; CH – Switzerland; CY – Cyprus; CZ – Czech Republic; DE – Germany; DK – Denmark; ES – Spain; FI – Finland; FR – France; GB – Great Britain; GR – Greece; HR – Croatia; IT – Italy; NL – The Netherlands; NO – Norway; PT – Portugal; RO – Romania; RS – Serbia; SE – Sweden; SK – Slovakia.
